# Impact of samarium on magnetic and optoelectronic properties of magnesium-based MgSm_2_X_4_ (X = S and Se) spinels for spintronics

**DOI:** 10.1371/journal.pone.0309388

**Published:** 2024-08-30

**Authors:** Nasir Rahman, Ahmed Azzouz-Rached, Mudasser Husain, Bashar M. Al-Khamiseh, Khmael M. Abualnaja, Ghaida Alosaimi, Vineet Tirth, Hassan Alqahtani, Ali Algahtani, Tawfiq Al-Mughanam, Soufyane Belhachi

**Affiliations:** 1 Department of Physics, University of Lakki Marwat, Lakki Marwat, KPK, Pakistan; 2 Physics Department, College of Science, UAE University, Al Ain, UAE; 3 Faculty of Exact Sciences, Magnetic Materials Laboratory, Djillali Liabes University of Sidi Bel-Abbes, Sidi Bel-Abbes, Algeria; 4 Institute of Condensed Matter and Material Physics Department of Physics, Peking University Beijing, Beijing, P. R. China; 5 MEU Research Unit, Middle East University, Amman, Jordan; 6 Faculty of Science, Department of Chemistry, Taif University, Taif, Saudi Arabia; 7 Mechanical Engineering Department, College of Engineering, King Khalid University, Abha, Asir, Kingdom of Saudi Arabia; 8 Research Center for Advanced Materials Science (RCAMS), King Khalid University, Guraiger, Abha, Asir, Kingdom of Saudi Arabia; 9 Department of Mechanical Engineering, Taibah University, Medina, Saudi Arabia; 10 Department of Mechanical Engineering, College of Engineering, King Faisal University, Al-Ahsa, Saudi Arabia; 11 Artificial Intelligence Laboratory for Mechanical and Civil Structures and Soil, University Center of Naama, Naama, Algeria; 12 Institute of Technology, University Center Salhi Ahmed of Naama, Naama, Algeria; National Research Centre, EGYPT

## Abstract

Investigating novel compounds has become necessary due to the need for sophisticated materials in optoelectronic devices and spintronics. Because of their unique properties, magnesium-based spinels MgSm_2_X_4_ (X = S and Se) are very promising for these applications. We used the spin-polarized PBEsol for structural properties and the PBEsol functional for mechanical behavior, both using the WIEN2k code. Both compounds’ stability in the magnetic and non-magnetic phases was validated by the Birch-Murnaghan equation of state, and their stability in the cubic phase was verified by the Born stability criterion. Their ductile character was shown by the computation of Pugh’s ratio and Poisson ratio. Both MgSm_2_S_4_ and MgSm_2_Se_4_ display metallic behavior in the spin-up channel and semiconducting behavior in the spin-down channel, indicating a half-metallic nature, according to TB-mBJ potential calculations. With total magnetic moments of 20 *μ*_B_, both materials showed ferromagnetic properties. Samarium ions contributed 5.27 *μ*_B_ for MgSm_2_S_4_ and 5.34 *μ*_B_ for MgSm_2_Se_4_. Furthermore, we computed optical parameters in the energy range of 0 to 15 eV, such as absorption, extinction coefficient, reflectivity, dielectric function, and refractive index. Our results demonstrate the potential of MgSm_2_X_4_ spinels for future technological developments by revealing their prospective optoelectronic and spintronic applications.

## 1. Introduction

Encouraging sustainable energy adoption, the decrease in natural energy resources and greenhouse gas emissions is spurred by the expected depletion of fossil fuel reserves in the next 50 years [1,2]. Spintronics expands microelectronics by manipulating and monitoring spins to process and store data, enhancing technological and scientific applications [3]. Spintronics in quantum technology boosts data storage and switching by manipulating electron spins, compressing magnetic chips. Since colossal magnetoresistance’s 1988 discovery, spin-up and spin-down states have revolutionized digital functionalities [4,5]. Magnetic fields can dramatically change the resistance of thin magnetic films, known as the colossal magnetoresistance effect, advancing memory technology. This spin manipulation has also revolutionized nano-sized, high-speed, and non-volatile-memory devices [6]. Half-metallic ferromagnets conduct in one spin channel but exhibit insulating or semiconducting behavior in the other [7]. Due to vacancies at the Fermi level in one spin channel, the density of states (DOS) at the Fermi level becomes fully spin-polarized [8]. Substituting several semiconductor materials with intrinsic band gaps can achieve properties suitable for spintronic ferromagnets (FM) [9]. Semiconductors are foundational to the functionality and advancement of a wide array of technologies that permeate nearly every aspect of modern life [10,11]. Semiconductor-based high-tech relies on a limited set of around ten active materials, including covalently bonded binary compounds such as diamond-like and zinc-blende structures, for a diverse range of electronic devices. Recent efforts to find new functionalities, such as semi-conductivity, transparent conductivity, or solar absorbance, have intensified. This response is driven by an increasing awareness of technological limitations within the traditional binary materials group [12–14]. In this regard, there has been considerable interest in the group of A_2_BX_4_ materials, where A and B are metallic elements, and X represents a chalcogen (O, S, Se, Te) [13,15,16]. With approximately 800 documented members [17], the A_2_BX_4_ group is known for its versatile physical properties. This group has potential applications in various fields, including transparent conductors (Cd_2_SnO_4_ and In_2_MgO_4_), thin film transistor materials (Zn_2_SnO_4_), lithium-ion battery materials (Mn_2_LiO_4_ and Co_2_LiO_4_), and thermoelectrics (Cr_2_CuSe_4_ and Cr_2_FeS_4_).

A_2_BX_4_ spinel materials, featuring metallic elements A and B along with chalcogen X (O, S, Se, Te), have emerged as promising contenders for diverse applications in various devices [18–21], owing to their remarkable properties [17,22–29]. Many researchers have extensively examined different spinel compounds for high-energy applications using the DFT approach. Using density functional theory (DFT), an investigation has been carried out on magnesium-based spinels, specifically MgX_2_O_4_ (X = Rh, Bi), for their potential in thermoelectric applications [16]. A study has been undertaken to examine the pressure-induced changes in the electronic and optical properties of MgA_2_Te_4_ (A = Sc, Y) [15]. A theoretical investigation was conducted on MgSc_2_Se_4_ and MgY2Se_4_, emphasizing the favorable applicability of MgSc_2_Se_4_ for magnesium-ion batteries owing to improved magnesium ion mobility in spinel compounds [26]. TmVO_4_, a wide-bandgap semiconductor (I41/amd space group), shows photocatalytic activity by utilizing 4f electron transitions of Tm3^+^ ions with VO_4_ tetrahedra, essential for organic contaminant degradation [30]. In a study, researchers developed a new visible-light-responsive bio-nanocomposite, ZnFe_12_O_19_-chitosan (ZF-C), to tackle issues like charge carrier recombination and enhance solar light absorption [31–37]. Shape- and size-controlled α-Fe_2_O_3_ nanoparticles were synthesized via a straightforward thermal treatment route using modified precursors [38]. Spinel compounds X_2_MgZ_4_ (X = Sc, Y; Z = S, Se) show promising optical applications with direct bandgaps and high absorption coefficients, as determined by the FP-LAPW + lo method [27]. Magnesium-based spinels MgX_2_O_4_ (X = Rh, Bi) were explored for thermoelectric applications using density functional theory (DFT) [16]. Theoretical exploration utilizing DFT has been conducted on magnesium-based spinels MgSc_2_X_4_ (X = S, Se), with the report extensively discussing the transport properties of these materials [28]. First-principles methods in DFT studied optoelectronic and transport properties of MgY_2_X_4_ spinel chalcogenides (X = S, Se). PBEsol + SOC determined lattice parameters and bulk modulus, while mBJ-LDA + SOC assessed electronic properties [29]. Computational techniques explored structural, electrical, and optical aspects of cubic spinel sulfides XSc_2_S_4_ (X = Zn, Mg, Be). Their favorable UV absorption and reflection make them suitable for photovoltaic and solar cell applications [30]. Employing DFT, the study investigated MgA_2_B_4_ spinel compounds (A = Sc, Y; B = S, Se), known for direct band gaps, with applications in optoelectronic devices. Density functional theory (DFT) investigated the spin-dependent structural, electronic, magnetic, and thermoelectric properties of MgSm_2_X_4_ (X = S, Se) [39]. Mechanical, spin-polarized electronic, and transport characteristics of MgSm_2_Y_4_ (Y = S, Se) spinels were investigated using first-principle calculations [40]. However, how these materials respond to light have not been investigated yet.

It prompts questions about unreported compounds and undiscovered ones yet to be explored, with the potential for revolutionary material functionalities. Keeping in view the literature and potential applications, we aim to explore MgSm_2_X_4_ (X = S, Se) to highlight their significance, exhibiting magnetic properties, direct band gap semiconducting traits, and half-metallic behavior for diverse applications.

## 2. Computational details

We conducted an investigation into the mechanical and thermoelectric properties of MgSm_2_X_4_ (X = S, Se) spinels, utilizing the WIEN2k code [41] based on density functional theory (DFT). The ground state lattice constants were determined using the spin-polarized Perdew–Burke–Ernzerhof version of the generalized gradient approximations (GGA–PBE) [42]. In the investigation, the mBJ potential, a modified Becke-Johnson potential [16,43,44], has been applied to analyze and explore the electronic properties of the system under study. This approach allows for a detailed examination of the electronic structure, offering insights into the behavior and characteristics of the electronic states within the material. Utilizing an 11 × 11 × 2 Monkhorst–Pack k-point grid, Brillouin zone (BZ) integration was performed, and the maximum value for the momentum orbital (*l*_max_) in expanding electronic wave functions into spherical harmonics within muffin-tin spheres was set to 10. Wave functions in the interstitial region were expanded using a plane-wave basis with a cutoff parameter RMT× *k*_max_ = 8 where *k*_max_ is the largest reciprocal lattice vector defining the plane wave basis set, and RMT is the smallest muffin-tin radii. In the calculations of total energy, the convergence criterion for energy was set to 10^−6^ Ry. For Mg, Sm, S, and Se, the respective muffin-tin radii (RMT) were 1.55, 2.14, 1.91, and 1.99 Bohr. The electronic orbital Mg: is 3s^2^, Sm: 6s^2^, S: 3s^2^ 3p⁴ and Se: 4s^2^ 4p⁴ were treated as valence states. The determination of elastic properties was carried out using the IRelast [45].

## 3. Results and discussion

### 3.1. Structural properties

**Fig 1:**
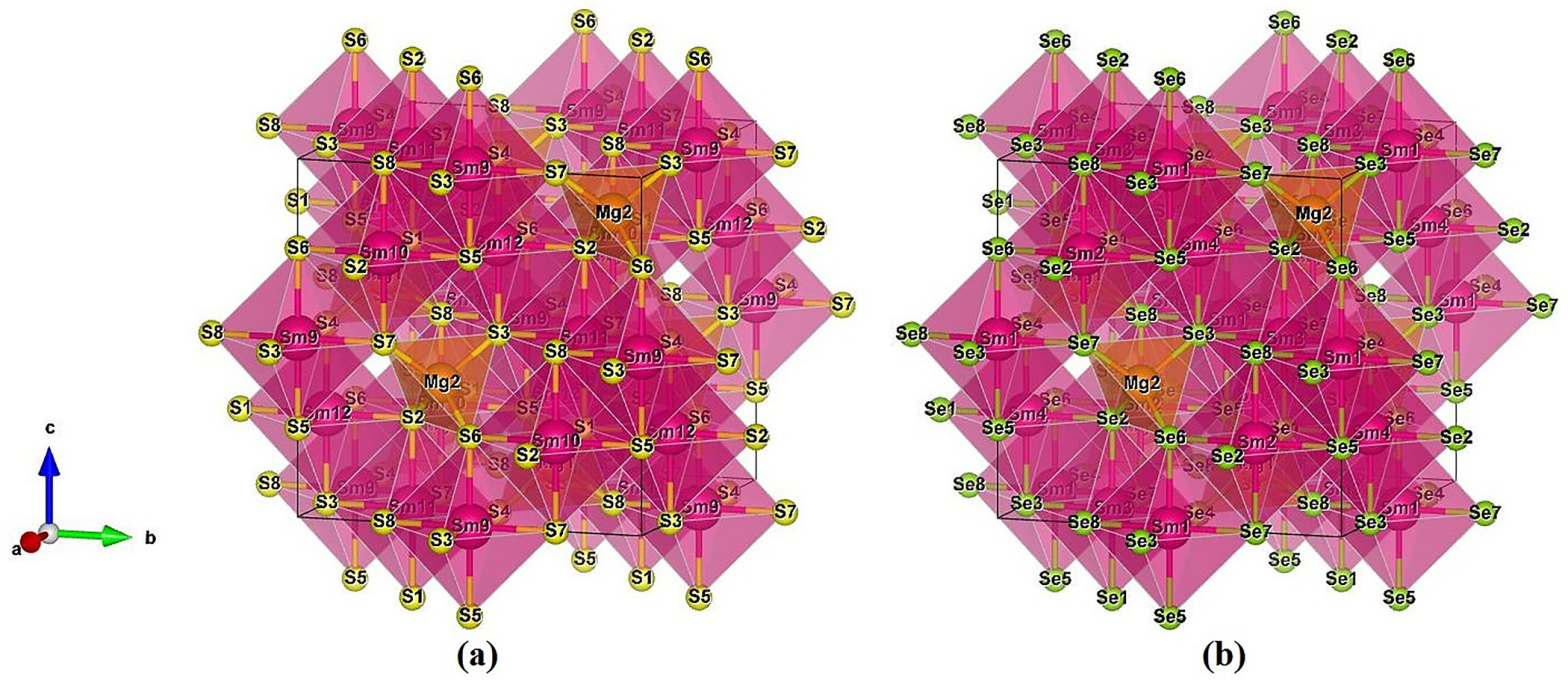
Crystal structure of MgSm_*2*_X_*4*_ (X = S and Se) (a) MgSm_*2*_S_*4*_ (b) MgSm_*2*_Se_*4*_.

The spinel structure of MgSm_2_X_4_ (where X = S and Se) crystallizes in a face-centered cubic arrangement with the space group 227-Fd-3m, shown in Fig 1. Sm occupies central positions in octahedra, and magnesium (Mg) takes central positions in tetrahedra. In both structures, oxygen (S, Se) fills 32 anionic positions. Octahedral sites are filled by 16d-positions of Sm cations, and Mg cations occupy the 8a-positions. In the spinel structure, the u-parameter (u_ideal_ = 0.25) ensures non-deviation along the (111) plane. The Fd−3m space group utilizes symmetry positions −3m or −4m, resulting in Wyckoff positions: (u, u, u) for (S, Se) atoms, (0.5, 0.5, 0.5) for Sm atoms, and (0.125, 0.125, 0.125) for Mg atoms.

**Fig 2:**
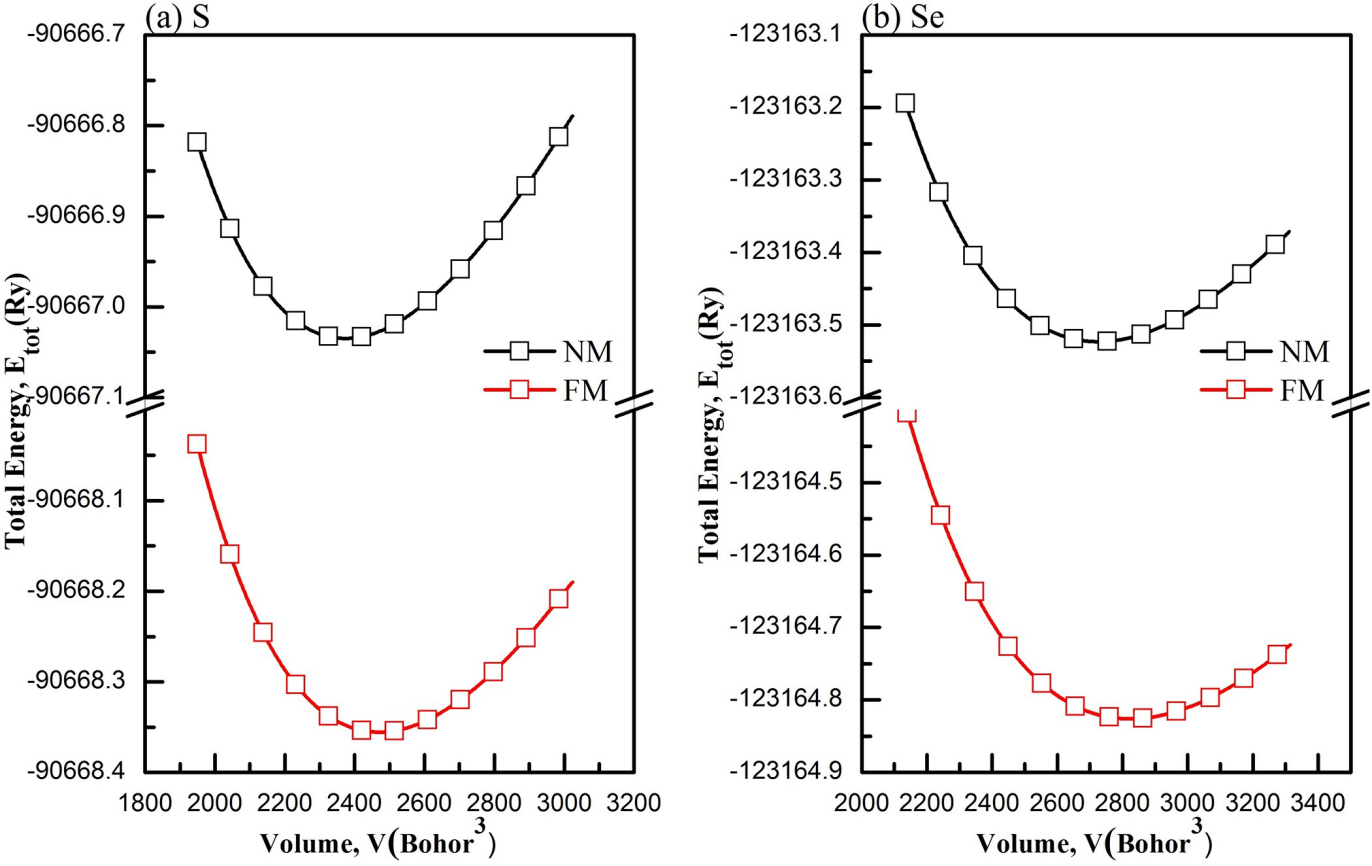
Total energy versus unit cell volume for the NM and FM configurations for (a) MgSm_*2*_S_*4*_ (b) MgSm_*2*_Se_*4*_compounds.

Murnaghan’s equation of state [46] is employed to calculate the bulk modulus (*B*), derivative of bulk modulus and the equilibrium cubic lattice parameter (*a*_0_), as outlined in Table 1. To pinpoint the stable magnetic configuration, we calculated the total energies of the two studied compounds for nonmagnetic (NM) and ferromagnetic (FM) configurations. Fig 2 displays the relationship between the calculated total energy (*E*) and unit cell volume (*V*) for both the magnetic configurations considered in both studied compounds. It is evident that both studied compounds exhibit stability in both magnetic states.

**Table 1:** The PBE-Sol GGA calculated lattice parameters such as lattice parameters (*a*_0_), bulk modulus (*B*), derivative of bulk modulus (*B*^/^), total energy (*E*_tot_) in both NM and FM magnetic phases.

Compounds		*a* (Å)	B (GPa)	*B* ^/^	*E*_tot_ (Ry)
**MgSm**_**2**_ **S**_**4**_	NM	11.20	60.98	3.91	-90667.034922
	FM	11.35	56.47	4.31	-90668.355604
**MgSm**_**2**_ **Se**_**4**_	NM	11.73	49.8898	4.1998	-123163.523159
	FM	11.87	46.5903	4.3065	-123165.826073

Using GGA based on the PBE proposal [26], structural parameters optimize, minimizing unit cell energy as shown in Fig 2. The diagram illustrates a decrease in unit cell energy with volume increase until reaching the ground state volume. Beyond this point, further increase indicates instability with increased system energy. First-principles calculations determine parameters based on the unit cell’s minimum energy state, reflecting the most stable configuration. The accuracy is verified by applying the Birch-Murnaghan EOS with adjustable parameters.

To further validate the structural stability and relaxation, the formation energies were calculated as follows:

$$E_{Form}^{MgSm2X4}=\frac{E_{total}^{MgSm2X4}-aE_{tot}^{Mg}-bE_{tot}^{Sm}-cE_{tot}^{X}}{a+b+c}\;\{X=S\;or\;Se\}$$ (1)

Here, $E_{Form}^{MgSm2X4}$ represents the formation energy of MgSm_2_X_4_, where X can be either S or Se. E_tot_ denotes the total energy of the bulk materials per unit cell, with ’a’, ’b’, and ’c’ representing the number of atoms of Mg, Sm, and X, respectively. The formation energy values indicate that MgSm_2_S_4_ has a formation energy of -2.528 eV/atom, while MgSm_2_Se_4_ has a formation energy of -1.928 eV/atom, underscoring the stability of these compounds, as indicated by their negative values.

**Fig 3:**
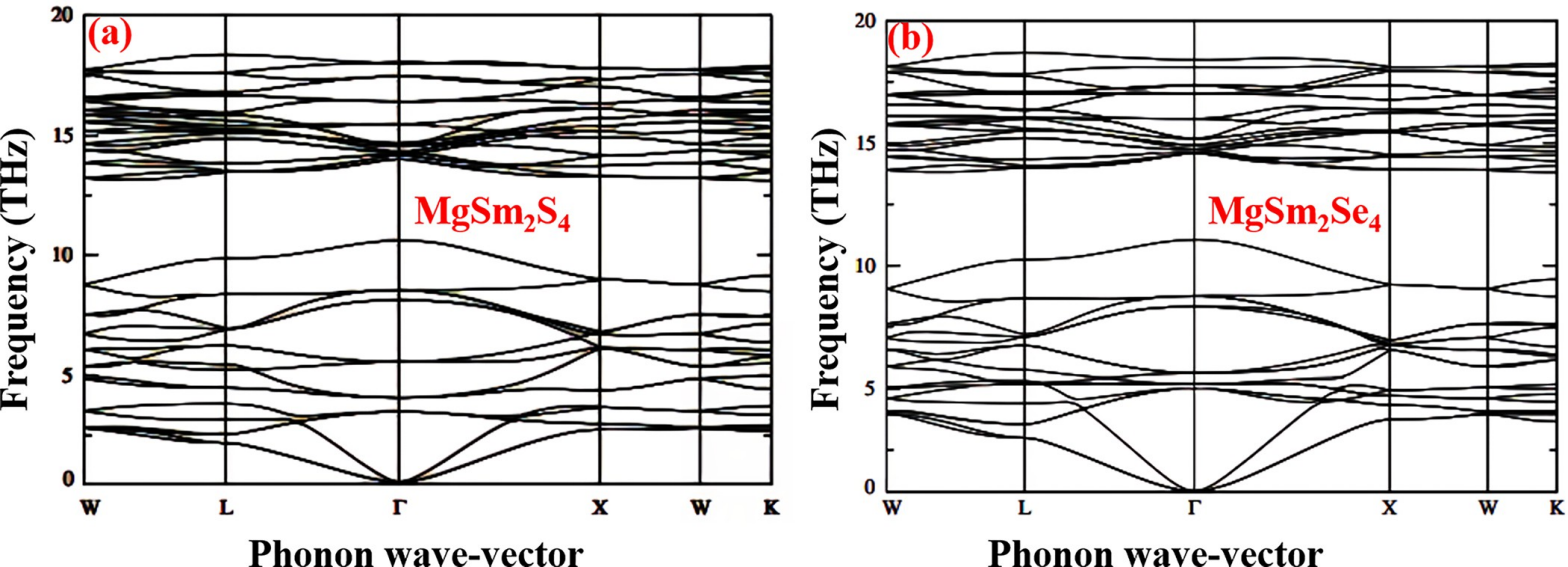
Phonon Band Structure of (a) MgSm_*2*_S_*4*_ (b) MgSm_*2*_Se_*4*_.

We investigated phonon band dispersions to understand a crystal lattice’s vibrational properties, which directly impact its mechanical and thermal behavior. For MgSm_2_X_4_ (X = S, Se) spinels, we calculated phonon dispersion curves (Fig 3). The dynamic stability of these compounds is confirmed by the absence of imaginary frequencies. Analyzing these dispersions clarifies the exchange of vibrational energy and atomic interactions. Understanding the optical and acoustic branches aids in comprehending thermal conductivity and potential thermoelectric applications.

### 3.2. Elastic properties

Elastic constants in materials play a vital role in determining strength, reflecting how materials respond to external forces. These values provide insights into bond characteristics, anisotropy, and structural stability. The determination of elastic constants utilized the IRelast package incorporated into the WIEN2k code. Elastic constants are pivotal for predicting material behavior in solid-state physics and materials science. They indicate stiffness, anisotropy, and responses to deformation. Rigid materials have higher elastic constants, and anisotropy is seen across crystallographic directions. These parameters help engineers to design structures with desired mechanical traits. In summary, elastic constants are foundational for tailoring materials and structures. Table 2 provides a comprehensive summary of the calculated elastic properties of the materials, using the equations outlined in the researchers’ study [44,47]. Confirming mechanical stability, Table 2 shows the studied materials meet required criteria. Maintaining mechanical stability in cubic crystal structures requires specific relationships among elastic constants. Summarized succinctly: *C*_11_—*C*_12_ and *C*_11_ + 2*C*_12_ should be positive, along with *C*_11_ and *B*.

**Table 2:** Calculated elastic parameters of MgSm_2_X_4_ (X = S, Se).

Elastic Parameters	MgSm_2_S_4_	MgSm_2_Se_4_
*C*_11_ (GPa)	89.10	92.27
*C*_12_ (GPa)	33.69	33.97
*C*_44_ (GPa)	41.55	41.55
*B* (GPa)	52.16	52.07
**A**	1.5	1.42
*E* (GPa)	86.40	88.80
*ʋ*	0.27	0.28
*B/G*	1.77	1.79
*C*_11_-*C*_12_ (GPa)	55.41	58.3
*G* (GPa)	35.30	36.52
*C*_11_-*C*_44_ (GPa)	47.55	50.72

MgSm_2_S_4_ has a *C*_11_ value of 89.10 GPa, while MgSm_2_Se_4_ has a higher *C*_11_ value of 92.27 GPa, indicating that MgSm_2_Se_4_ is harder. The study emphasizes the significance of elastic anisotropy "*A*" in the formation of micro-cracks. In materials that are fully isotropic, "*A*" equals 1; any deviation indicates anisotropy. The observed deviation in both materials confirms their distinct anisotropic behavior. Various features differentiate ductile and brittle behavior in materials. Cauchy’s pressure, derived from *C*_11_ and *C*_44_ [46,48], categorizes based on *C*_11_-*C*_44_. Positive difference indicates ductility, and in this study, both materials exhibit it: 47.55 GPa for MgSm_2_S_4_ and 50.72 GPa for MgSm_2_Se_4_, confirming ductile nature. To determine the forces present in the material, Poisson’s ratio (ν) was calculated among elastic parameters. As a result, Poisson’s ratio (*ν*) ranged from 0.25 to 0.5 [49]. The determined value of was established at 0.26, indicating the brittleness or ductility of any compounds. Our computed (*ν*) value confirms that MgSm_2_X_4_ (X = S and Se) exhibits ductile characteristics. Furthermore, to gauge a material’s ductility or brittleness, the Pugh ratio (*B/G*) is used, with a critical threshold at 1.75. Higher B/G ratios indicate enhanced ductility [50]. In this case, both compounds surpass the critical threshold, with MgSm_2_S_4_ at 1.77 and MgSm_2_Se_4_ at 1.79. Stability, anisotropy, ductility, and significant resistance to cracking are exhibited by the analyzed compounds.

### 3.3. Electronic properties

**Fig 4:**
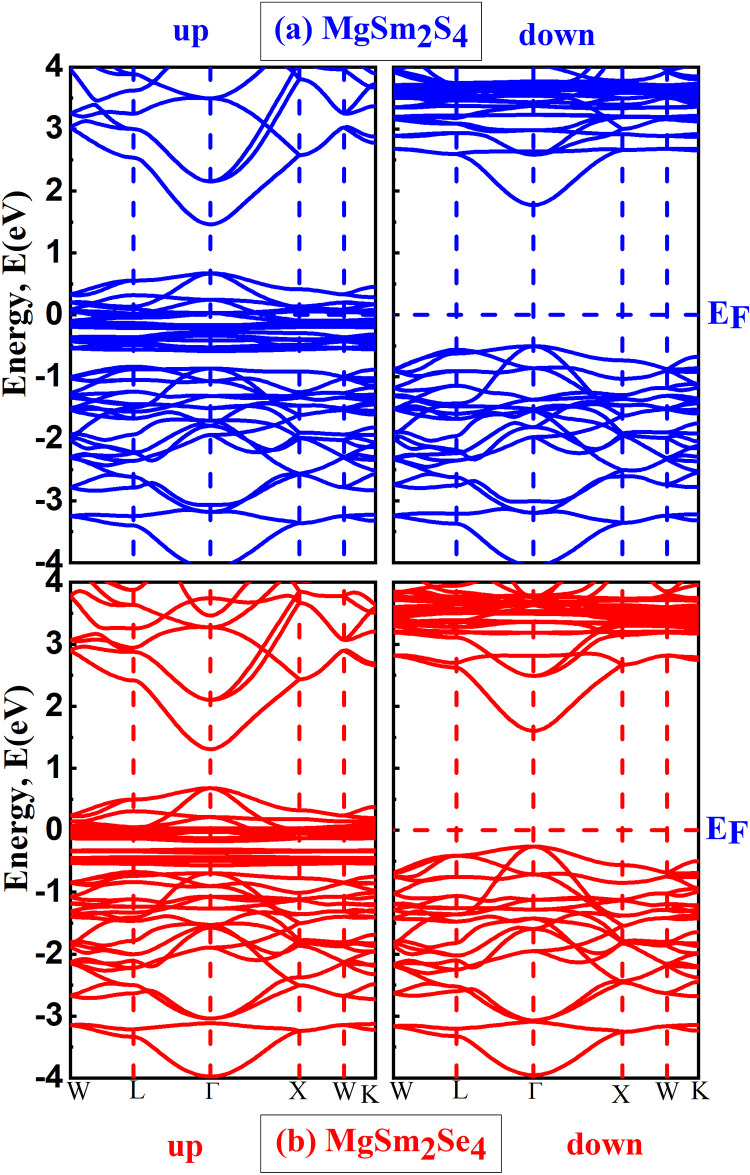
Band structure profile for both spin up and spin down channel of (a) MgSm_*2*_S_*4*_ (b) MgSm_*2*_Se_*4*_.

Understanding band structure is crucial for predicting and interpreting the electronic, optical, and thermal properties of materials, facilitating the design and optimization of electronic devices. Shown in Fig 4 is the illustrations of the band structure of MgSm_2_X_4_ (X = S, Se). Both of our materials demonstrate half metallic behavior. It can be seen that in the spin up configuration both of these compounds while in spin down both are semiconducting. A half-metallic material, exhibiting metallic behavior in spin-up and semiconducting behavior in spin-down, is intriguing for its unique electronic structure. In the spin-up state, it acts as a metal with high conductivity, while in the spin-down state, it resembles a semiconductor with a distinct band gap, restricting electron flow under specific conditions. The distinctive electronic structure of half-metallic materials is appealing for applications in spintronics and magnetic materials, offering opportunities to develop advanced electronic devices that combine the advantages of both metallic and semiconducting behaviors. Researchers explore these materials for their potential to create efficient and versatile components in emerging technologies.

In Fig 4A for MgSm_2_S_4_, it is evident that in the spin-up channel, the bands overlap, indicating a metallic nature. Conversely, in the spin-down channel, there is a 2.27 eV gap between the valence band and conduction band, signifying the semiconductor’s direct band gap nature from Γ- Γ symmetry point. Fig 4B illustrates MgSm_2_Se_4_, where in the spin-up channel, the overlapping bands suggest a metallic nature. Conversely, the spin-down channel exhibits a 1.86 eV gap between the valence band and conduction band, indicating the semiconductor’s direct band gap nature from Γ- Γ symmetry point. Table 3, demonstrates the band gap values both in spin up and spin down channel.

**Table 3:** Band gap values both in spin up and spin down channel.

Material	Spin configuration	Bandgap (eV)
**MgSm**_**2**_ **S**_**4**_	Up	00
	down	2.27
**MgSm**_**2**_ **Se**_**4**_	Up	00
	Down	1.86

**Fig 5:**
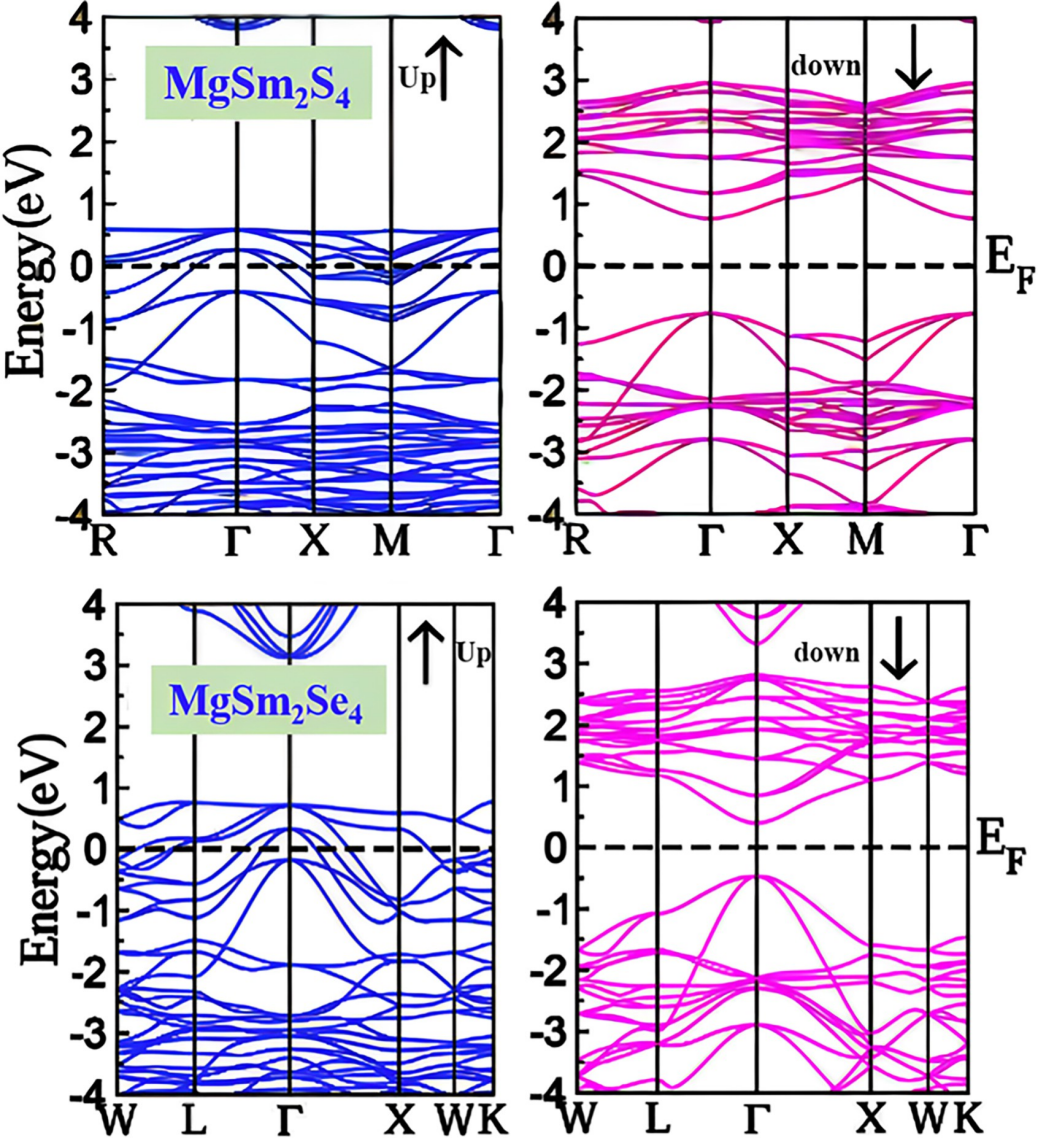
Band structure of MgSm_2_X_4_ (X = S, Se) with the inclusion of Spin-orbit coupling.

Due to its heavy atomic mass and 4f electrons, the Sm (Samarium) atom has a significant effect on the spin-orbit coupling (SOC) in MgSm_2_S_4_ and MgSm_2_Se_4_ as shown in Fig 5. Its high atomic number augments SOC through stronger relativistic effects. The partially filled 4f orbitals of Sm contribute to SOC by interacting with the surrounding electronic environment, which results in band structure modifications, particularly near the Fermi level, which alter the electronic and magnetic properties, such as band splitting or new spin-polarized states.

**Fig 6:**
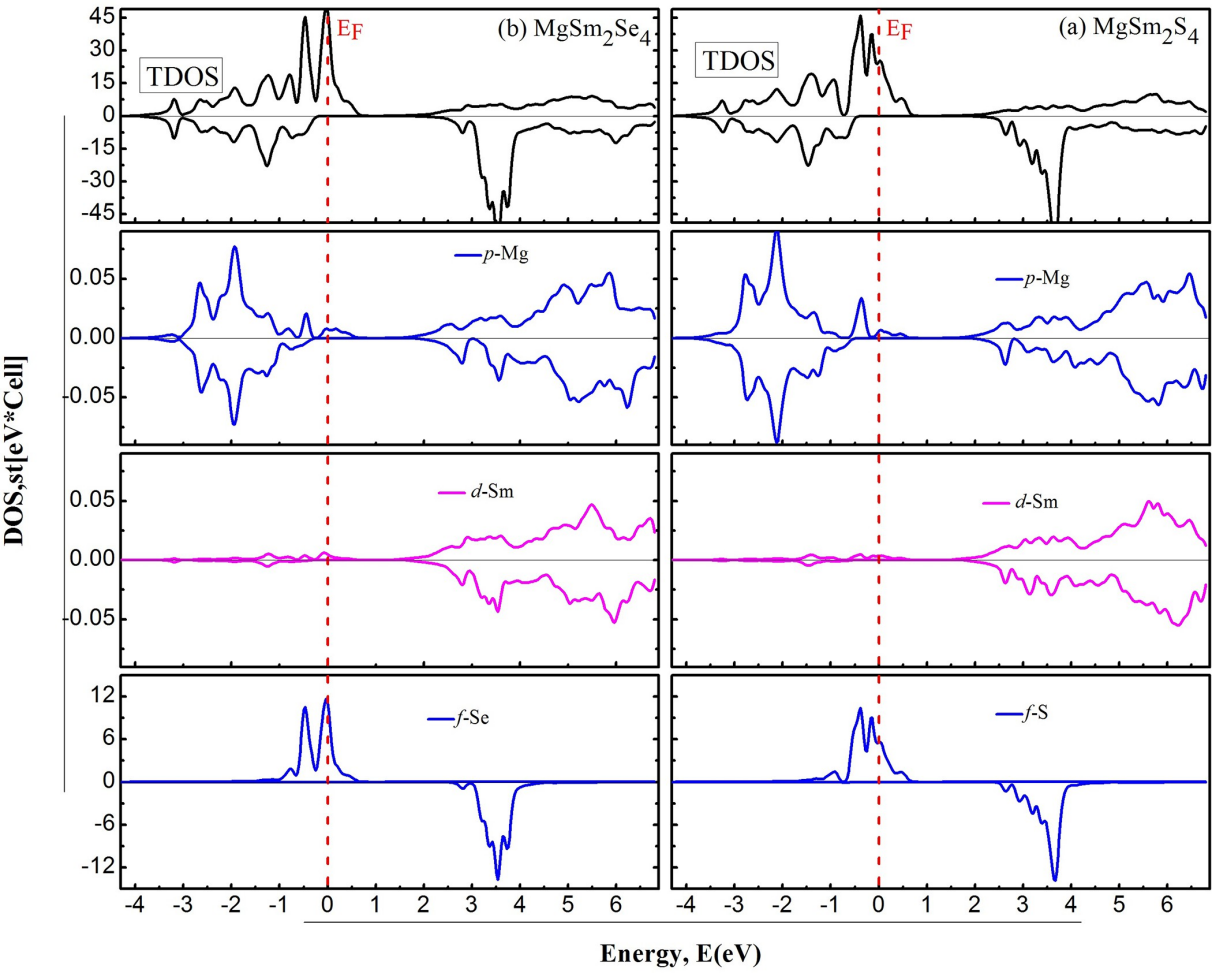
Calculated total and partial (DOS) for (a) MgSm_*2*_S_*4*_ and (b) MgSm_*2*_Se_*4*_.

To delve deeper into the electronic properties, a Density of States (DOS) analysis is conducted to examine the contributions of various states. In Fig 6A and 6B, for a thorough examination of band structures, both the partial and total densities of states (DOS) are graphed, focusing on the p state of Mg, d state of Sm, and f state of Se to enhance the interpretation. In MgSm_2_Se_4_ (depicted in Fig 6B), magnesium (Mg) primarily influences the valence band through its p states, occupying energy levels from 0 to -3 eV. Additionally, Mg contributes to the conduction band with its p states, prevalent in the energy range of 2 to 6 eV. Samarium (Sm) plays a role in the conduction band, utilizing its d states within the 2 to 6 eV energy range. Selenium (Se) participates in the valence band via its f states, spanning from -1 to 1.8 eV, proximate to the Fermi level. Furthermore, Se’s f states contribute to the conduction band, manifesting in the energy range of 2.5 to 4 eV. These energy intervals offer valuable insights into the electronic structure and distinctive orbital contributions of each element within MgSm_2_Se_4_.

In MgSm_2_S_4_, depicted in Fig 4A, one can observe contributions from p-states of magnesium (p-Mg), d-states of samarium (d-Sm), and f-states of sulfur (f-S). DOS calculations further validate that in both of our compounds, the metallic nature is apparent in the spin-up channel, where overlapping bands are observed. Conversely, the spin-down channel reveals a gap between the valence and conduction bands, indicating the semiconductor’s band gap nature. Due to their special optoelectronic qualities, emerging materials including perovskite materials and two-dimensional (2D) semiconductors (such as graphene and molybdenum disulfide) are demonstrating significant potential. High carrier mobility and robust light-matter interactions are possible with 2D semiconductors, which is advantageous for next-generation transistors and photodetectors. Perovskite materials are becoming more and more popular because of their high efficiency and adjustable optical characteristics, which make them excellent options for cutting-edge solar cells and light-emitting gadgets.

### 3.4. Magnetic properties

The magnetic moments, both atomic and total, calculated for the ferromagnetic MgSm_2_S_4_ and MgSm_2_Se_4_ are presented in Table 4. Observing the total magnetic moment is crucial, as it is the sum of magnetic moments from all atoms within the unit cell and the interstitial region. The overall magnetic moment for MgSm_2_S_4_ is 20 *μ*_B_ indicating that the compound has a significant magnetic character. The magnesium (Mg) contribution to the magnetic moment is 0 *μ*_B_. This suggests that the magnetic moment associated with Mg is negligible or not present. Samarium (Sm) contributes 5.27 *μ*_B_ to the total magnetic moment. This positive value indicates that Sm is a significant contributor to the magnetic character of the compound. A positive magnetic moment of 5.27 *μ*_B_ for samarium (Sm) indicates that the magnetic moments associated with individual samarium atoms in the compound MgSm_2_S_4_ are aligned in the same direction. This alignment contributes to the overall magnetic character of the compound, making it more likely to exhibit ferromagnetic or strongly magnetic behavior. Positive values typically suggest a parallel alignment of magnetic moments, which is a characteristic feature of ferromagnetic materials. The sulfur (S) contribution to the magnetic moment is -0.13 *μ*_B_. The negative value implies a diamagnetic or antiferromagnetic contribution, counteracting the overall magnetic moment. The interstitial region contributes -0.04 *μ*_B_ to the total magnetic moment. Similar to sulfur, the negative value suggests a diamagnetic or antiferromagnetic influence. In summary, the compound MgSm_2_S_4_ demonstrates a substantial total magnetic moment, largely influenced by the positive contribution from samarium, suggesting the likelihood of exhibiting ferromagnetic characteristics.

**Table 4:** Calculated Total (*μ*_B_), Mg (*μ*_B_), Sm (*μ*_B_), S/Se (*μ*_B_) and Interstitial (*μ*_B_).

Compounds	Total (*μ*_B_)	Mg (*μ*_B_)	Sm (*μ*_B_)	S/Se (*μ*_B_)	Interstitial (*μ*_B_)
**MgSm**_**2**_ **S**_**4**_	20	0.00	5.27	-0.13	-0.04
**MgSm**_**2**_ **Se**_**4**_	20	0.00	5.34	-0.16	-0.12

Similarly as shown in Table 4, the overall magnetic moment for MgSm_2_Se_4_ is recorded at 20 μB, indicating a substantial magnetic character within the compound. Magnesium (Mg) makes no significant contribution to the magnetic moment, registering at 0.00 *μ*_B_. This implies that the magnetic moment associated with Mg is either negligible or absent. Samarium (Sm) plays a crucial role by contributing 5.34 *μ*_B_ to the total magnetic moment. This positive value suggests that the magnetic moments associated with individual samarium atoms align in the same direction, pointing towards potential ferromagnetic behavior. Selenium (Se) contributes -0.16 *μ*_B_ to the magnetic moment, with a negative value indicating a diamagnetic or antiferromagnetic influence, opposing the overall magnetic moment. The interstitial region contributes -0.12 *μ*_B_ to the total magnetic moment. Similar to selenium, the negative value implies a diamagnetic or antiferromagnetic influence. MgSm_2_Se_4_ displays a significant total magnetic moment, mainly shaped by the positive influence of samarium, suggesting the potential for ferromagnetic behavior. The interplay of contrasting contributions from magnesium, selenium, and the interstitial region introduces complexity, with selenium’s negative contribution offsetting samarium’s positive effect. In conclusion, the magnetic behavior of MgSm_2_Se_4_ is steered by samarium, and the intricate interplay of diverse contributions adds complexity to the compound’s magnetic characteristics. Furthermore, MgSm_2_X_4_ exhibit antiferromagnetic behavior where the magnetic moments of Sm ions are arranged in antiparallel orientations to each other. This alignment is complemented by S/Se ions and interstitial sites, which also exhibit antiparallel alignment with the Sm moments. Together, these interactions result in a total magnetic moment of 20 *μ*_B_, characteristic of its antiferromagnetic nature.

### 3.5. Optical properties

**Fig 7:**
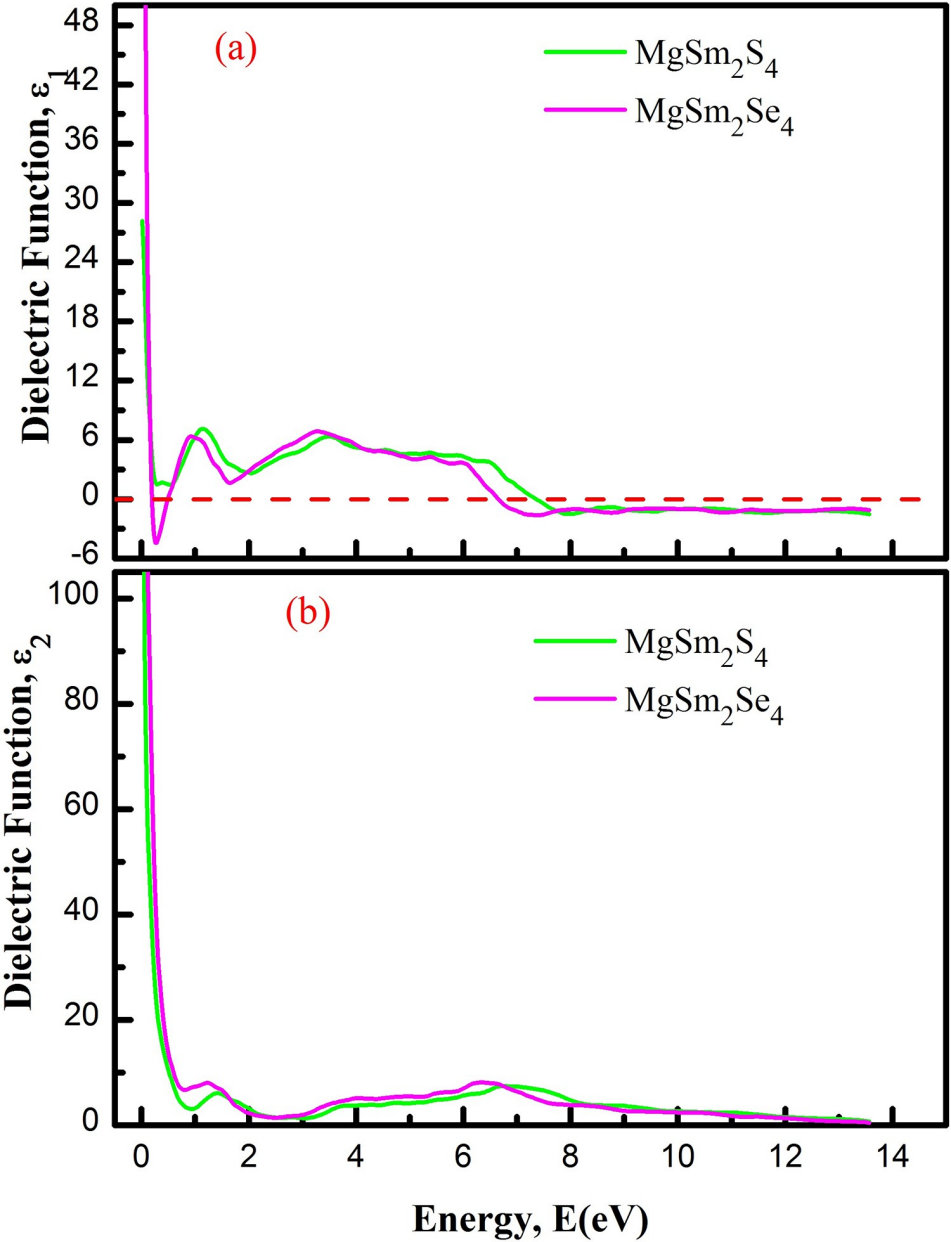
Dielectric function of MgSm_*2*_X_*4*_ (X = S, Se) (a) Real Part (b) Imaginary Part.

A material’s suitability for solar cells and optoelectronic devices depends on its interaction with light, causing transitions within or between bands. In optoelectronic applications, indirect band gap semiconductors, like MgSm_2_X_4_ (X = S, Se) double perovskites, play a crucial role. These materials involve transitions that include emission or absorption of phonons, essential for radiative processes in light emission and absorption. The indirect band gap in MgSm_2_X_4_ makes them suitable for such radiative processes, prompting an analysis of their optical characteristics in the energy range of 0–15 eV. In Fig 7A, the calculated real part of the dielectric function ε_1_(ω) for MgSm_2_X_4_ (X = S, Se) spinals is shown. This parameter reflects the material’s dispersive behavior and describes its response to incident electromagnetic radiations [51,52]. Differences in bandgap values result in varied static real dielectric function parts. MgSm_2_S_4_, with larger bandgaps (in spin down configuration), shows lower values, especially near the bandgap. This signifies robust transitions between electronic states, as the dielectric function indicates the material’s reaction to incident electromagnetic radiation. A negative peak at 0.25 eV in the dielectric function indicates absorption, suggesting electronic transitions or excitations in the material within the visible to mid-infrared spectrum. In MgSm_2_S_4_, a peak value of 7.26 is noted at 1.11 eV, and for MgSm_2_Se_4_ peak value of 6.12 is observed at 0.95 eV, demonstrated in Fig 7A. Moreover, in the visible range, these spinal compounds show significant response.

**Fig 8:**
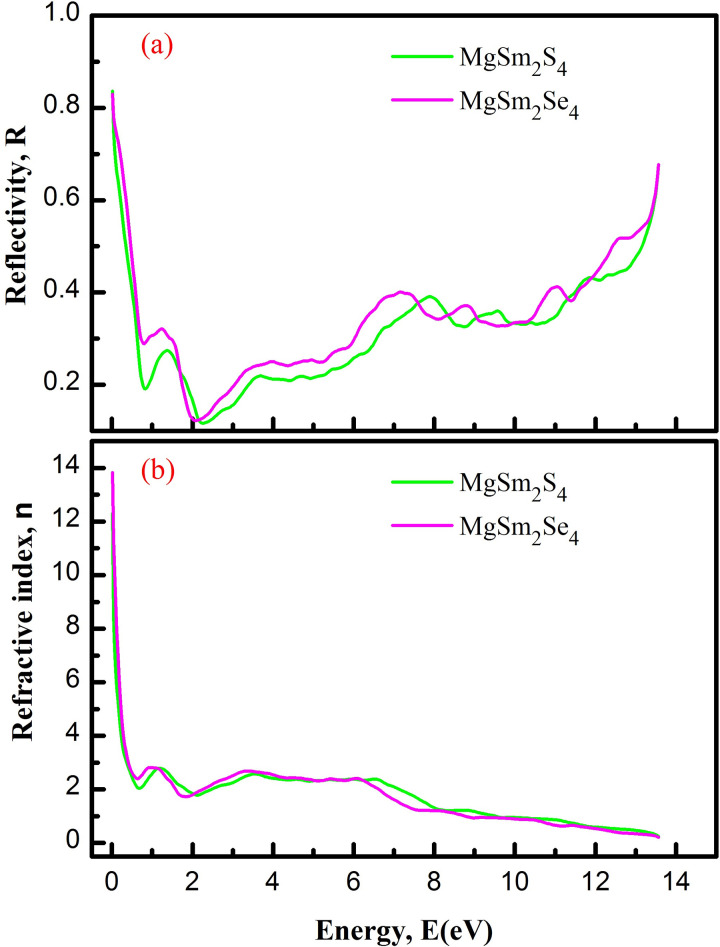
(a) Reflectivity (b) Refractive Index of MgSm_*2*_X_*4*_ (X = S, Se).

The imaginary part of the dielectric function, ε_2_(ω), quantifies the material’s absorption of incident radiation by measuring energy loss as waves traverse. Fig 5B shows ε_2_(ω) thresholds for MgSm_2_S_4_ and MgSm_2_Se_4_: 2.27 eV and 1.86 eV, respectively, aligning closely with the compounds’ band gaps. This highlights the accuracy of the current calculations, as both MgSm_2_X_4_ compounds display finely varying peaks. R(ω) characterizes surface photon reflection, indicating the proportion of reflected electromagnetic radiation [53]. Fig 8A depicts the computed R(ω). Nevertheless, R(0) follows the pattern observed in ε₁(0). Moreover, R(ω) values remain below 25% in the visible range, suggesting that both MgSm_2_X_4_ (X = S, Se) spinals can effectively absorb visible photons. Therefore, these materials are recommended for potential use in optoelectronic devices. Higher reflectivity, indicating an increased ability of the materials to bounce back incident light, is observed as the reflectivity of both the MgSm_2_X_4_ compounds increases from 2 to 14 eV. This suggests that the materials become more effective at reflecting electromagnetic radiation within that energy range, potentially impacting their optical properties and applications.

**Fig 9:**
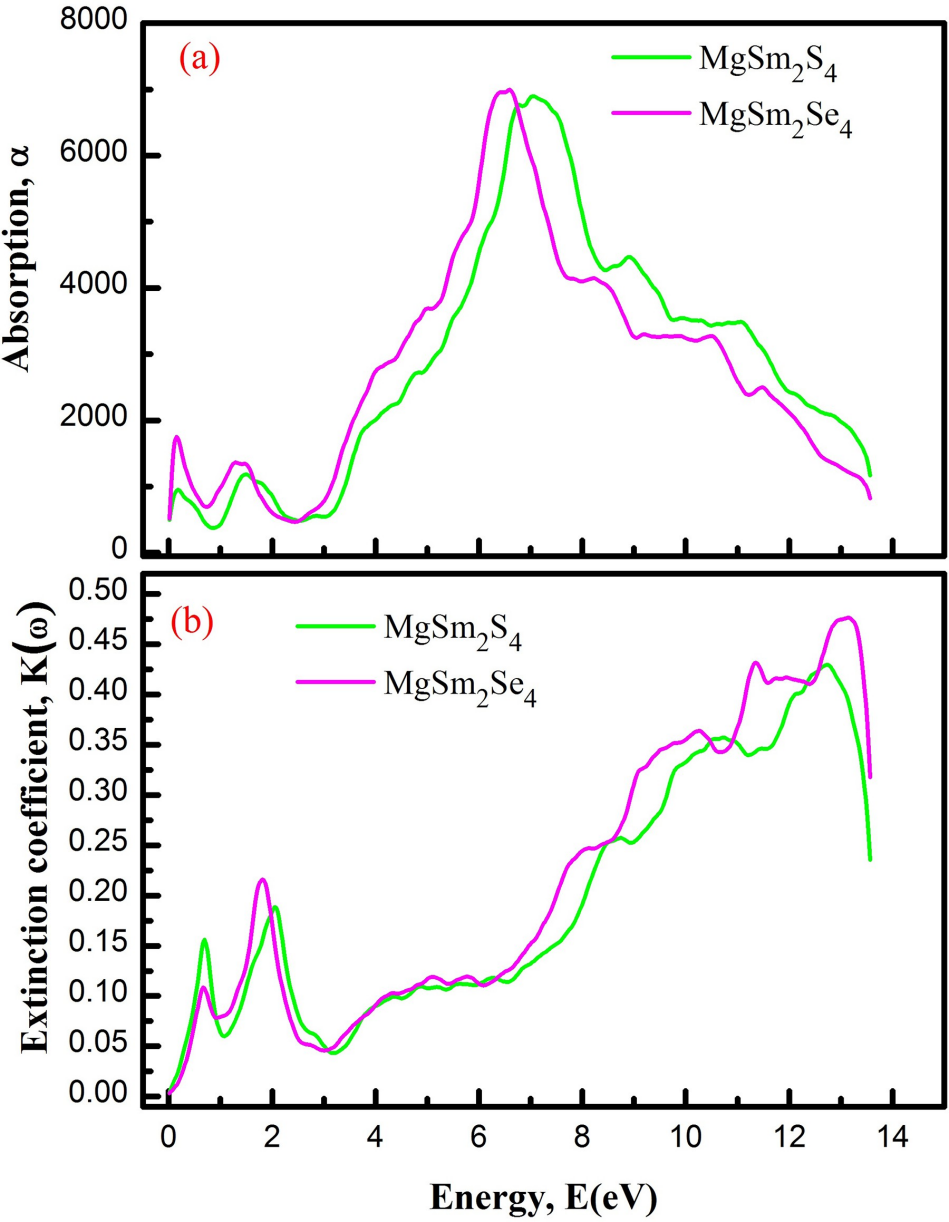
(a) Absorption (b) Extinction coefficient of MgSm_*2*_X_*4*_ (X = S, Se).

The refractive index gauges how light slows or bends in a material, with Fig 8B illustrating changes relative to incident photon energy. The refractive index (n) is 2.27 for MgSm_2_S_4_ and 1.81 for MgSm_2_Se_4_, indicating a decrease transitioning from S to Se, with intermittent peaks observed in the 0 to 12 eV energy range. Se larger size compared to S influences atom spacing in the lattice, altering the refractive index and light interaction. Intermittent peaks in the 0 to 12 eV range suggest electronic transitions, contributing to observed refractive index changes. Fig 9A illustrates the light absorption spectrum α(ω) of MgSm_2_X_4_ compounds (where X is S and Se) for clarity on light absorption. Beyond the threshold energy, the ε_2_ curve sharply rises, peaking at 6400 for MgSm_2_S_4_ at 8.1 eV and 6500 for MgSm_2_Se_4_ at 7.7 eV incident photon energy. These peaks indicate significant absorption in this energy range. The absorption threshold in Fig 9A closely aligns with ε_2_, while the electronic energy gap (*E*_g_) slightly exceeds these thresholds, likely due to the exclusion of exciton effects in the calculation.

Fig 9B illustrates that the extinction coefficient κ(ω) represents the reduction in the amplitude of the incoming electric field oscillation. For both examined materials, κ(ω) starts at the fundamental band gap, indicating the absorption of incident radiations. The presence of Mg, Sm, and the specific arrangement of atoms in the crystal lattice contribute to charge transfer transitions, contributing to absorption features.

## 4. Conclusion

We employed the WIEN2k code to explore the structural, elastic, optoelectronic, and magnetic properties of magnesium-based MgSm_2_X_4_ (X = S and Se) spinels. Utilizing PBEsol for mechanical behavior and spin-polarized PBEsol for structural properties, we investigated MgSm_2_X_4_ (X = S and Se). The Birch-Murnaghan equation of state confirmed the structural stability of both these compounds in both magnetic and non-magnetic phases. The Born stability criterion confirmed stability in the cubic phase for the investigated spinels, and their ductile behaviors were determined through Pugh’s ratio and Poisson ratio calculations. By examining electronic properties through TB-mBJ potential calculations, it was evident that both compounds exhibited metallic behavior in the spin-up channel and semiconducting behavior in the spin-down channel, signifying a half-metallic nature. Both materials displayed ferromagnetic characteristics, with the total magnetic moments summing up to 20 (*μ*_B_). This behavior was attributed to the presence of samarium contributing 5.27 (*μ*_B_) for MgSm_2_S_4_ and 5.34 (*μ*_B_) for MgSm_2_Se_4_. Optical properties, including absorption, extinction coefficient, reflectivity, dielectric function, and refractive index, were calculated in the energy range of 0 to 15 eV, highlighting the importance of these two compounds in optoelectronic devices. The findings suggest that the examined spinels hold promise as potential candidates for spintronics applications.
