# Correction: Impact of samarium on magnetic and optoelectronic properties of magnesium-based MgSm_2_X_4_ (X = S and Se) spinels for spintronics

**DOI:** 10.1371/journal.pone.0316763

**Published:** 2024-12-30

**Authors:** Nasir Rahman, Ahmed Azzouz-Rached, Mudasser Husain, Bashar M. Al-Khamiseh, Khamael M. Abualnaja, Ghaida Alosaimi, Vineet Tirth, Hassan Alqahtani, Ali Algahtani, Tawfiq Al-Mughanam, Soufyane Belhachi

The fifth author’s name is spelled incorrectly. The correct name is: Khamael M. Abualnaja. The correct citation is: Rahman N, Azzouz-Rached A, Husain M, Al-Khamiseh BM, Abualnaja KM, Alosaimi G, et al. (2024) Impact of samarium on magnetic and optoelectronic properties of magnesium-based MgSm_2_X_4_ (X = S and Se) spinels for spintronics. PLOS ONE 19(8): e0309388. https://doi.org/10.1371/journal.pone.0309388.
